# Etiologies underlying subtypes of long-standing type 2 diabetes

**DOI:** 10.1371/journal.pone.0304036

**Published:** 2024-05-28

**Authors:** Riad Bayoumi, Muhammad Farooqi, Fatheya Alawadi, Mohamed Hassanein, Aya Osama, Debasmita Mukhopadhyay, Fatima Abdul, Fatima Sulaiman, Stafny Dsouza, Fahad Mulla, Fayha Ahmed, Mouza AlSharhan, Amar Khamis

**Affiliations:** 1 College of Medicine, Mohammed Bin Rashid University of Medicine and Health Sciences, Dubai, UAE; 2 Dubai Diabetes Center, Dubai Health, Dubai, UAE; 3 Endocrinology Department, Dubai Hospital, Dubai Health, Dubai, UAE; 4 Pathology Department, Dubai Hospital, Dubai Health, Dubai, UAE; National Healthcare Group, SINGAPORE

## Abstract

**Background:**

Attempts to subtype, type 2 diabetes (T2D) have mostly focused on newly diagnosed European patients. In this study, our aim was to subtype T2D in a non-white Emirati ethnic population with long-standing disease, using unsupervised soft clustering, based on etiological determinants.

**Methods:**

The Auto Cluster model in the IBM SPSS Modeler was used to cluster data from 348 Emirati patients with long-standing T2D. Five predictor variables (fasting blood glucose (FBG), fasting serum insulin (FSI), body mass index (BMI), hemoglobin A1c (HbA1c) and age at diagnosis) were used to determine the appropriate number of clusters and their clinical characteristics. Multinomial logistic regression was used to validate clustering results.

**Results:**

Five clusters were identified; the first four matched Ahlqvist et al subgroups: severe insulin-resistant diabetes (SIRD), severe insulin-deficient diabetes (SIDD), mild age-related diabetes (MARD), mild obesity-related diabetes (MOD), and a fifth new subtype of mild early onset diabetes (MEOD). The Modeler algorithm allows for soft assignments, in which a data point can be assigned to multiple clusters with different probabilities. There were 151 patients (43%) with membership in cluster peaks with no overlap. The remaining 197 patients (57%) showed extensive overlap between clusters at the base of distributions.

**Conclusions:**

Despite the complex picture of long-standing T2D with comorbidities and complications, our study demonstrates the feasibility of identifying subtypes and their underlying causes. While clustering provides valuable insights into the architecture of T2D subtypes, its application to individual patient management would remain limited due to overlapping characteristics. Therefore, integrating simplified, personalized metabolic profiles with clustering holds greater promise for guiding clinical decisions than subtyping alone.

## 1 Introduction

Diabetes is a heterogenous disease [1, 2], with well-defined categories such as type 1 diabetes (T1D), latent autoimmune diabetes in adults (LADA), and monogenic types. The remaining patients are pooled together under T2D. However, patients with T2D present with a wide spectrum of clinical symptoms, and a range of variables that have a direct impact on glucose homeostasis. Patients may develop T2D at an early age or late in life [3]. They may be lean, overweight, obese, or morbidly obese [4–6]. The disease may be characterized by failure of insulin secretion or insulin resistance or both; may progress either rapidly or slowly and may be mild or severe. It may lead to one or more complications with a variety of outcomes [2]. Consequently, the clinical paradigm of one-size-fits-all leads to management and treatment failures in many patients [7]. Thus, there is a need to subtype T2D into distinct, well-defined groups [8] to better understand the underlying mechanisms, treatment responses, and prognoses associated with the disease. Several studies have attempted to identify T2D subtypes in patients of white European origin using various approaches, such as statistical clustering algorithms, clinical characteristics, genetics, and biomarkers [9–17]. T2D clustering was also replicated in other ethnic groups [18–22]. In most of these studies, subtyping of T2D was based on newly diagnosed patients [9–17] with a few exceptions where the temporal stability of clusters was tested in patients with short-term disease [13, 16, 23]. Few studies have attempted T2D subtyping in long-standing disease [3, 21–24] to avoid the different rates of disease progression and the impact of complications [3, 25–29].

Most studies have also used unsupervised K-means hard clustering methods with definitive assignment of data points to single clusters and reported distinct subtypes [9, 11–13, 18–22]. However, some other studies employed unsupervised soft clustering methods with the likelihood of data points belonging to more than one cluster and reported T2D subtypes with considerable overlap [14–17]. In this study, we continue to investigate the heterogeneity of T2D by cluster analysis of Emirati Arab T2D patients with long-standing disease, using unsupervised soft clustering algorithms.

## 2 Methods

### 2.1 Study design

This retrospective, cross-sectional, non-interventional study was conducted at the Dubai Diabetes Centre and Dubai Hospital of Dubai Health, Dubai, UAE. Dubai Diabetes Centre is dedicated to the specialized care of patients with diabetes. Dubai Hospital is a specialty hospital equipped with 600 beds and provides surgical and medical facilities. Both follow the American Diabetes Association Standards of Medical Care for Diabetes [2].

This study was approved by the Dubai Scientific Research Ethics Committee of Dubai Health Authority. Approval No. DSREC-12/2019-05 was issued on January 23, 2020. Further IRB extensions were granted by DSREC on 28^th^ April 2021 and 10^th^ May 2022. Written informed consent was obtained from patients during the face-to-face interviews. All relevant clinical and laboratory data were obtained from the Dubai Health Information System “SALAMA”. Information gathered was anonymized to maintain patient privacy and confidentiality. Clinical management and treatment protocols, all laboratory methods, radiographic imaging, and data obtained from the “SALAMA” hospital information system adhered to relevant Dubai Health regulations and guidelines and conformed to the provisions of the Declaration of Helsinki (as revised in Fortaleza, Brazil, October 2013).

### 2.2 Patients

We aimed to collect enough data to identify genuine underlying disease clusters and avoid creating random ones. We anticipated our analysis to yield 4–5 subgroups, each containing a minimum of 20–30 observations. This ensured sufficient data points to effectively define the characteristics of each cluster.

A cohort of 348 Emirati patients with T2D were recruited from a database of 620 patients who underwent random screening between January 24th, 2020 and December 31st, 2022, at the outpatient departments of the Dubai Diabetes Centre and Dubai Hospital. The selected patients had complete data for all clustering parameters. Patients were tested for GAD antibodies (ELISA Test Kit; Demeditec Diagnostics, GmbH, Germany) to exclude T1D and LADA. The selected patients ranged in age from 18 to 87 years and included 167 men and 181 women. They had an average T2D duration of 14 years and at least two co-morbidities or complications. Each patient had been on two or more medications (metformin, thiazolidines, SGLT2 inhibitors, and GLP-1 agonists) for a minimum of two years. Patients with conditions causing secondary diabetes were excluded. For each patient, the clinical and laboratory data were obtained from the SALAMA electronic health record system used by all health facilities affiliated to Dubai Health. The recorded medical history, comorbidities, and complications of the disease were confirmed through face-to-face interviews with the patients.

### 2.3 Statistics

#### 2.3.1 Cluster analysis

IBM SPSS Modeler (IBM North America, New York, USA) was used for clustering analysis. The software provides several machine-learning algorithms that can be used for classification, regression, clustering, and anomaly detection. These algorithms are based on artificial neural networks and deep learning.

The Auto Cluster model in IBM SPSS Modeler was utilized in the exploratory phase to determine the optimal clustering solution for the dataset comprising 348 Emirati patients with long-standing T2D. Five T2D variables (FBG, FSI, BMI, HbA1c and age at diagnosis) were standardized and employed as predictors to identify the suitable number of clusters and their characteristics. To mitigate the influence of confounding factors, such as comorbidities and complications associated with the long-standing T2D phenotype, the predictor variables were limited to those five parameters that directly or indirectly influence the disease’s pathophysiology. FBG and FSI levels are two crucial etiological factors that directly reflect the underlying pathophysiology of T2D. We used them in the initial exploratory technique for grouping the data. Furthermore, FBG and FSI were used in the explanatory process to assess peripheral insulin resistance (HOMA-IR) and/or impaired insulin secretion (HOMA-B), that served as descriptors of the clusters, not as part of the clustering process itself.

The BMI is used as a predictor for T2D because it is strongly linked to insulin resistance, a key factor in T2D pathophysiology. While HbA1c isn’t strictly an etiological variable for T2D, it plays a pivotal role in its diagnosis, monitoring, and prognosis. It is a valuable tool for providing long-term insights into glycemic control and the risk of developing long-term complications. Age at diagnosis of T2D reflects the cumulative effect on metabolic dysfunction and the duration of risk factors for complications. Early-onset T2D is associated with a more aggressive course and higher risk of complications, while late-onset disease is usually more benign.

The Auto Cluster model operates as a Bayesian Network Model for classification purposes. It sequentially employs three unsupervised soft clustering algorithms:

1. A two-steps process: The initial step involves a single pass through the data to condense the raw input into a manageable set of subclusters. Subsequently, a hierarchical clustering method is utilized in the second step to progressively merge these subclusters into larger clusters. The two-step approach offers the advantage of automatically estimating the optimal number of clusters.
2. The K-means clustering algorithm: This method defines a fixed number of clusters and iteratively assigns records to clusters while adjusting the cluster centers until further refinement does not enhance the model. Unlike predictive modelling, k-means employs unsupervised learning to uncover patterns within the input fields.
3. The Kohonen algorithm: This generates a neural network capable of clustering the dataset into distinct groups. Once fully trained, similar records should be closed together on the output map, while dissimilar records will be positioned farther apart. This process also aids in determining an appropriate number of clusters.

Following sufficient iteration for each model, the Auto Cluster will produce a Silhouette index, with the model exhibiting the highest index being selected. The Auto Cluster node prioritizes algorithms and allocates data points into the relevant clusters accordingly. For both the two-steps and the K-means algorithms the same Silhouette Index of 0.64 was generated.

The Bayesian network assigns probabilities of membership to the participants in the five identified clusters. Each participant is represented as a node in the network by predictor variables and becomes an additional node that influences the main cluster assignment node. Conditional probability distributions model probabilistic dependencies, allowing computation of the likelihood of a participant belonging to a specific cluster, given their observed variables. Conditional probability distributions enable accurate inferences and yield probabilities of membership for each participant in one or more clusters. Therefore, the model computes the overlap of an individual within multiple clusters, as displayed in a heatmap [S1 Table].

The model also assigns membership and measures the degree of overlap between clusters using the silhouette coefficient. A high silhouette coefficient indicates that the data point is well-matched to its own cluster and poorly matched to neighboring clusters, with less overlap between the clusters. The silhouette coefficient ranges from -1 to 1. A value of 1 indicates that the data point fits perfectly into a single cluster, while a value of -1 indicates that the data point does not fit into any cluster. A value of 0 indicates that the data point is equally suited for two or more clusters. To highlight the clinical characteristics of clusters, data for individuals with a silhouette coefficient of 1.0, and/or probability of 1.0, on the Bayesian Network were selected, as they sat in the non-overlapping apices of clusters. They exhibited the highest degree of dysfunction in the etiological processes governing cluster membership. As one lowers the silhouette coefficient values in a sliding scale below 1.0, the degree of overlap decreases and the number of individuals in the non-overlapping apices of clusters increases but their clinical homogeneity and discreetness drops.

Principal component analysis was used to identify the linear combinations of the original variables that explained most of the variance in the data and to extract the features that were most correlated with the clustering variables. Principal component analysis has also been used to visualize the dataset and help in identifying clusters, as it transforms the dataset into a lower-dimensional space where the clusters are more easily separated.

#### 2.3.2 Multinomial logistic regression

Multinomial logistic regression in SPSS 29 was used in the explanatory phase of the study to validate the results of clustering performed by the Auto Cluster model in IBM SPSS Modeler and predict the probability of categorical dependent variables (Clusters 1–5), given a set of the five independent predictor variables (FBG, FSI, BMI, HbA1c, and age at diagnosis). The model was refined using a maximum likelihood procedure to determine the values of the model parameters that maximized the likelihood of the observed data. The relationship between the predictors and category of the dependent variable was modelled using the log-odds of each category relative to a reference category (Cluster 5). The outcome of the multinomial logistic regression model was a set of regression coefficients (B) for each predictor variable. The coefficients were then used to rank the importance of the predictors in each cluster. The odds ratio (OR) is a measure of the association between a predictor variable and a cluster, calculated by exponentiating the B value. The larger the B value, the stronger the association. An OR > 1 indicated that the predictor variable was associated with an increased risk of the outcome falling into a particular cluster relative to the reference category (Cluster 5).

#### 2.3.3 HOMA assessment

FBG, FSI and other chemistry assays were performed using a Cobas 6000 Analyzer (Hoffmann La Roche Diagnostics, CA, US). Insulin resistance (IR) and β-cell dysfunction (B) were evaluated by homeostatic model assessment for IR (HOMA-IR) and β-cell dysfunction (HOMA-B) [30]. The HOMA indices were derived from FBG and fasting serum insulin levels using the following equations:

$$HOMA-IR=FBG\times \frac{insulin}{22.5}$$ (1)

$$HOMA-B=20\times \frac{insulin}{\left(FBG-3.5\right)}$$ (2)

The higher the HOMA-IR, the greater the peripheral resistance to insulin, while the lower the HOMA-B, the greater the β-cell dysfunction. Generally, a HOMA-IR value < 1 indicates optimal insulin sensitivity. Levels above 1.9 indicate early resistance; levels above 2.9 indicate significant resistance. A HOMA-B value < 100 indicates β-cell dysfunction.

## 3 Results

### 3.1 Demographics

The mean age of the 348 Emirati patients with T2D was 56 years, and the mean duration of diabetes was 14 years. The mean BMI was 31 and the mean age at diagnosis was 42 years. Gender-wise demographic characteristics are shown in Table 1.

**Table 1:** Demographic characteristics of 348 Emirati T2D patients selected for subtyping of the disease.

	Men (N = 167)	Women (N = 181)	P-value
**Age (years)**	56.34 (9.81)	56.30 (11.27)	0.598
**BMI (Kg/m** ^**2**^ **)**	30.19 (5.16)	32.26 (6.00)	<0.001
**Waist hip ratio**	1.0 (0.06)	0.94 (0.08)	<0.001
**Age at diagnosis (years)**	41.43 (10.32)	42.34 (11.00)	0.396
**Duration of diabetes (years)**	14.91 (8.10)	13.96 (8.18)	0.247

All values are shown as Mean (±Standard Deviation).

Owing to the long duration and chronicity of T2D, considerable deterioration in the metabolic profiles of selected patients was observed. Of all patients, 90 (26%) had HOMA-IR > 3.0, indicating peripheral insulin resistance, while 140 (40%) had HOMA-B < 100, indicating pancreatic secretion dysfunction. The remaining 118 (34%) exhibited both pathophysiological dysfunctions. The prevalence of comorbidities and complications observed were also high, with hypertension at 62%, peripheral neuropathy at 53%, retinopathy at 33%, and coronary artery disease at 15% (Table 2). In most patients, at least two comorbidities or complications of diabetes were observed.

**Table 2:** Prevalence of comorbidities and complications of T2D in 348 Emirati patients recruited for subtyping of the disease.

	Total (N = 348)	Men (N = 167)	Women (N = 181)	P-value
**Retinopathy**	117 (34)	67 (40)	50 (28)	0.009
**Cataract**	33 (10)	13 (8)	20 (11)	0.196
**Glaucoma**	14 (4)	8 (5)	6 (3)	0.335
**CKD**	43 (12)	21 (13)	22 (12)	0.517
**CAD**	51 (15)	35 (21)	16 (9)	0.001
**History of Stroke**	26 (8)	16 (10)	10 (.6)	0.111
**Hypertension**	221 (64)	111 (67)	110 (61)	0.161
**PAD**	14 (4)	8 (5)	6 (3)	0.330
**Peripheral Neuropathy**	189 (54)	88 (53)	101 (56)	0.318

CKD- chronic kidney disease; CAD- coronary artery disease; PAD- peripheral artery disease

All values are shown as Number of patients (percentage)

### 3.2 Cluster analysis

Results of the cluster analysis of the cohort of 348 Emirati patients with T2D with long-standing disease, are shown in Table 3. No significant differences in cluster results were observed between male and female T2D patients. Therefore, results were reported for the total cohort throughout the manuscript. Five Clusters were identified in this study. The first four matched Ahlqvist et al [11] subgroups. Cluster 1 had severe insulin-resistant diabetes (SIRD) in 8% of patients. Cluster 2 had severe insulin deficient diabetes (SIDD) in 16%. Cluster 3 had mild age-related diabetes (MARD) in 25%. Cluster 4 had mild obesity-related diabetes (MOD) in 21%. A fifth new subtype of mild early onset diabetes (MEOD) was identified in 30% of mostly lean patients.

**Table 3:** Results of clustering analysis of 348 Emirati T2D patients using IBM SPSS modeler.

Clusters	Number of patients, N (%)			Average Silhouette Score
	Total	with no overlap between clusters	with overlap between clusters	
**SIRD**	27 (8)	25 (7)	2 (1)	+ 0.126
**SIDD**	57 (16)	40 (11)	17 (5)	- 0.272
**MARD**	86 (25)	24 (7)	62 (18)	- 0.275
**MOD**	73 (21)	23 (7)	50 (14)	- 0.219
**MEOD**	105 (30)	39 (11)	66 (19)	- 0.110
**Total**	348 (100)	151 (43)	197 (57)	

SIRD- severe insulin-resistant diabetes; SIDD- severe insulin deficient diabetes; MARD- mild age-related diabetes; MOD- mild obesity-related diabetes; MEOD- mild early-onset diabetes.

A Silhouette Index of 1.0 indicates no overlap and <1.0 indicates overlap between clusters.

All values are shown as Number of patients (percentage).

**Fig 1:**
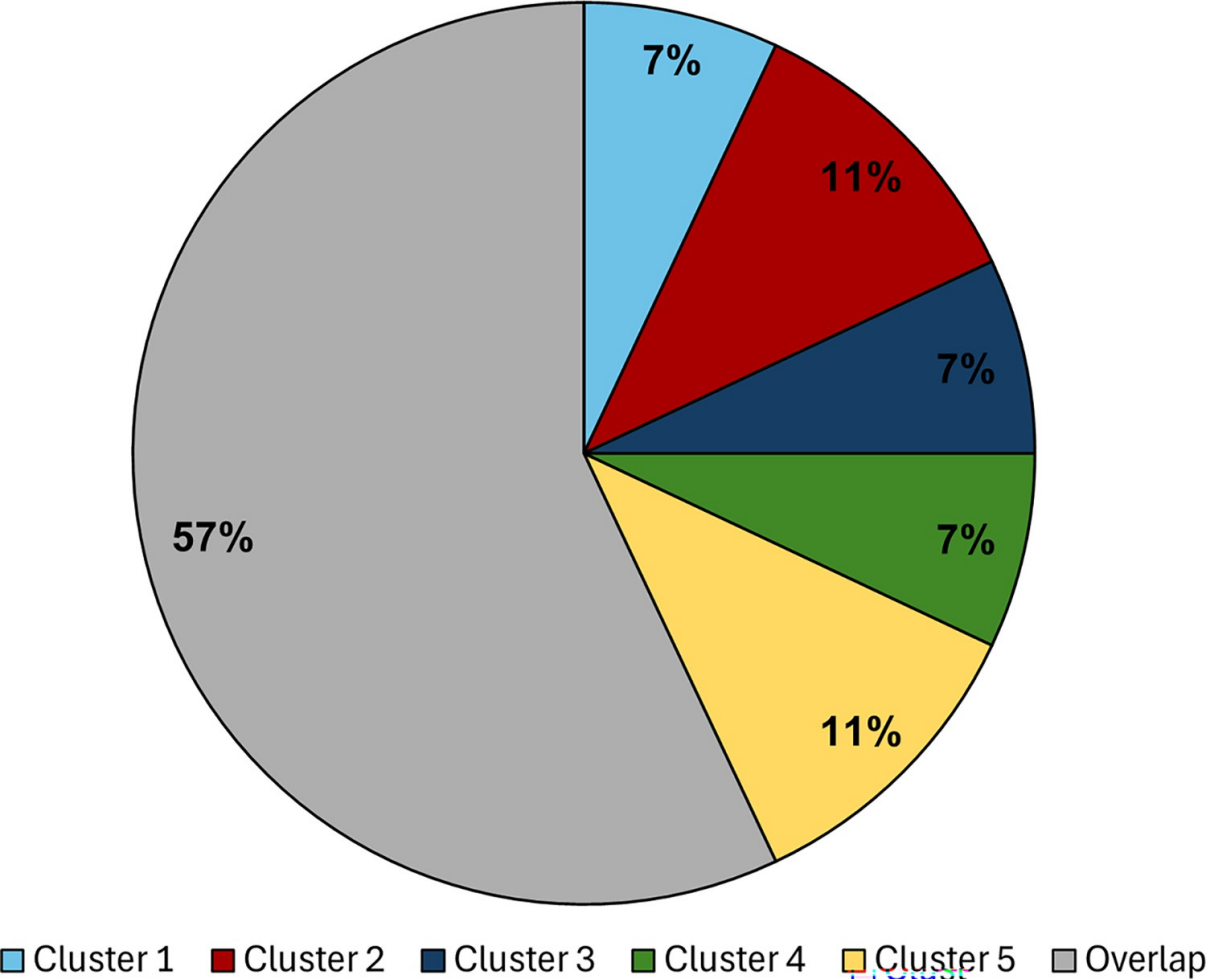
Distribution of five clusters, among 348 Emirati T2D patients, with and without overlap: Distribution of patients is shown as percentage of total patients (N = 348) into overlapping clusters in grey, and percentage of patients exclusive to 1 cluster as light blue (Cluster 1- SIRD), red (Cluster 2- SIDD), dark blue (Cluster 3- MARD), green (Cluster 4- MOD), and yellow (Cluster 5- MEOD).

However, there was extensive overlap between clusters. Cluster 1 (SIRD), with a positive average silhouette score, did not significantly overlap with any of the other clusters. The other four clusters, with negative average silhouette scores, seemed to overlap extensively. There were 151 patients (43%) with membership in cluster peaks with no overlap, as confirmed by a Silhouette Index and Bayesian probability of 1.0 (Table 3). The remaining 197 patients (57%) showed extensive overlap between clusters confirmed by a Silhouette Index and Bayesian probability of <1.0 (S1 Table) with individuals appearing in two or more clusters (Fig 1).

**Fig 2:**
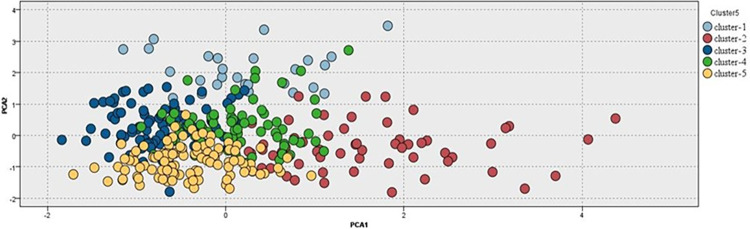
Display of principal component analysis of clusters of 348 Emirati T2D patients: Each circle represents a patient in a cluster. The colors represent each cluster as light blue (Cluster 1- SIRD), red (Cluster 2- SIDD), dark blue (Cluster 3- MARD), green (Cluster 4- MOD), and yellow (Cluster 5- MEOD).

Principal component analysis (PCA) was used to visualize the dataset and identify the five T2D clusters, as it transformed the dataset into a lower-dimensional space where the clusters were more easily separated (Fig 2). Multinomial logistic regression was used to explain the relationship between predictor variables and categorical outcomes (clusters) and validate clustering results. We identified predictor variables that were significantly associated with the clusters and quantified the strength of these associations. The higher the regression coefficients (B) and the odds ratio (OR), the stronger the contribution to the cluster (Table 4). The model coefficients ranked the importance of the predictor variables in each cluster [S2 Table].

**Table 4:** Estimation of the contribution of the five independent variables (Age at diagnosis, body mass index, fasting insulin, fasting blood glucose and HbA1c) as predictors for T2D clusters using multinomial logistic regression.

Cluster	Predictors	Regression Coefficient (B)	P-value	Odds Ratios [95%CI]
**SIRD**	*Fasting Insulin*	*0*.*904*	*< 0*.*001*	*2*.*47 [1*.*66–3*.*66]*
** **	BMI	0.741	< 0.001	2.1 [1.154–2.86]
** **	Age at diagnosis	0.355	< 0.001	1.43 [1.83–1.72]
** **	FBG	0.012	0.534	1.01 [0.98–1.05]
** **	HbA1c	-0.201	0.781	0.82 [0.20–3.36]
**SIDD**	*HbA1c*	*2*.*823*	*< 0*.*001*	*18*.*83 [6*.*12–46*.*26]*
** **	BMI	0.323	0.002	1.38 [1.12–1.70]
** **	Insulin	0.222	< 0.001	1.25 [1.10–1.43]
** **	FBG	0.092	< 0.001	1.10 [1.06–1.14]
** **	Age at diagnosis	0.056	0.28	1.057 [0.28–1.06]
**MARD**	*Age at diagnosis*	*0*.*385*	*< 0*.*001*	*1*.*47 [1*.*33–1*.*63]*
** **	BMI	0.246	0.005	1.28 [1.08–1.52]
** **	Insulin	0.206	< 0.001	1.23 [1.12–1.34]
** **	FBG	-0.014	0.151	0.99 [0.97–1.01]
** **	HbA1c	-0.809	0.05	0.45 [0.20–1.0]
** MOD**	*BMI*	*0*.*834*	*< 0*.*001*	*2*.*30 [1*.*86–2*.*85]*
** **	Insulin	0.113	0.01	1.12 [1.03–1.22]
** **	HbA1c	0.183	0.537	1.20 [0.67–2.15]
** **	Age at diagnosis	0.022	0.565	1.02 [0.95–1.10]
** **	FBG	0.008	0.366	1.01 [0.99–1.03]

SIRD- severe insulin-resistant diabetes; SIDD- severe insulin deficient diabetes; MARD- mild age-related diabetes; MOD- mild obesity-related diabetes; BMI- body mass index; FBG- fasting blood glucose; HbA1c- Hemoglobin A1c.

The predictors with highest contribution are italicized for each cluster.

### 3.3 Cluster characteristics

The pathophysiological characteristics, and laboratory data of the five clusters identified in the cohort of 348 Emirati patients with long-standing T2D is shown in Table 5. Characteristics of 4 clusters matched that of Ahlqvist et al [11] subgroups. Cluster 5 patients had a novel subtype of mild early-onset diabetes (MEOD) in mostly lean patients.

**Table 5:** The pathophysiological characteristics of T2D subtypes in Emirati patients with long standing disease in Dubai, UAE.

	SIRD N = 27 (8%)	SIDD N = 57 (16%)	MARD N = 86 (25%)	MOD N = 73 (21%)	MEOD N = 105 (30%)	Total N = 348 (100%)
**Age (years)**	55.2 (10.2)	52.4 (9.7)	64.6 (8.4)	53.4 (9.4)	54.0 (10.0)	56.3 (10.6)
**Age at diagnosis (years)**	45.5 (11.3)	37.9 (8.0)	53.1 (8.0)	38.3 (8.5)	36.5 (7.7)	41.9 (10.7)
**Duration of diabetes (years)**	9.7 (6.4)	14.5 (8.2)	11.6 (6.2)	15.1 (9.0)	17.5 (8.1)	14.4 (8.1)
**BMI (Kg/m** ^**2**^ **)**	34.3 (4.1)	32.0 (6.4)	29.9 (3.7)	37.0 (4.8)	27.2 (3.2)	31.3 (5.7)
**HOMA-IR**	16.0 (6.4)	7.5 (4.9)	4.3 (2.5)	4.3 (2.6)	2.7 (1.6)	5.3 (4.8)
**HOMA-B**	244 (260)	33 (19)	109 (103)	78 (76)	58 (45)	85 (111)
**FBG (mg/dL)**	152 (39)	224 (63)	123 (28)	136 (31)	129 (32)	146 (52)
**RBG (mg/dL)**	185 (72)	228 (99)	140 (55)	174 (70)	168 (79)	173 (80)
**Insulin (mU/ml)**	42.1 (10.0)	13.1 (7.1)	13.9 (6.5)	12.4 (7.1)	8.5 (4.6)	14.0 (10.6)
**HbA1c (%)**	7.3 (1.00)	10.4 (2.1)	6.7 (0.8)	7.4 (1.1)	7.1 (0.9)	7.6 (1.7)
**Blood Urea (mg/dL)**	29 (9)	28 (9)	35 (26)	31 (14)	29 (10)	31 (16)
**Serum Creatinine (mg/dL)**	0.80 (0.24)	0.79 (0.68)	0.84 (0.41)	0.78 (0.30)	0.73 (0.24)	0.79 (0.00)
**eGFR (mL/min)**	95 (18)	101 (15)	86 (22)	96 (23)	99 (18)	95 (20)
**Urine Albumin (mg/L)**	44 (68)	102 (335)	79 (217)	124 (538)	24 (56)	73 (302)
**Urine creatinine (mg/dL)**	136 (87)	117 (71)	105 (67)	108 (91)	98 (76)	108 (78)
**Albumin/Creatinine Ratio**	52 (134)	164 (727)	143 (695)	189 (654)	36 (80)	117 (551)
**Blood Cholesterol (mg/dL)**	151 (36)	171 (39)	153 (39)	166 (42)	156 (37)	159 (39)
**HDL (mg/dL)**	45 (12)	43 (10)	48 (13)	48 (11)	47 (11)	47 (12)
**LDL (mg/dL)**	79 (27)	96 (36)	79 (34)	90 (35)	84 (31	85 (34)
**Triglycerides (mg/dL)**	158 (89)	155 (82)	128 (61)	143 (89)	125 (79)	137 (79)
**ALT (IU/L)**	24 (11)	25 (14)	26 (27)	25 (24)	23 (18)	25 (21)
**AST (IU/L)**	22 (8)	29 (30)	22 (11)	22 (12)	22 (15)	23 (17)
**ALP (IU/L)**	74 (21)	92 (28)	77 (24)	82 (32)	71 (20)	79 (26)

SIRD- severe insulin-resistant diabetes; SIDD- severe insulin deficient diabetes; MARD- mild age-related diabetes; MOD- mild obesity-related diabetes; MEOD- mild early-onset diabetes; BMI- body mass index; HOMA-IR- homeostatic model assessment for insulin resistance; HOMA-B- homeostatic model assessment of β-cell Function; FBG- fasting blood glucose; RBG- random blood glucose; HbA1c- Hemoglobin A1c; eGFR- estimated glomerular filtration rate; HDL- high-density lipoprotein; LDL- low-density lipoprotein; ALT- alanine aminotransferase; AST- aspartate aminotransferase; ALP- Alkaline Phosphatase.

All values are shown as Mean (±Standard Deviation).

### 3.4 Etiological processes governing T2D subtypes

To highlight the major etiological processes governing membership of subtypes, we selected data of patients at the non-overlapping apices of cluster distributions (N = 151), confirmed by a Silhouette Index ≥ 1.0 (Table 6).

**Table 6:** Data of the major etiological processes governing T2D subtypes in subsets of individuals at the non-overlapping apices of distribution of clusters.

	SIRD (N = 25)	SIDD (N = 40)	MARD (N = 24)	MOD (N = 23)	MEOD (N = 39)
**Age (years)**	55.2 (10.5)	50.8 (10.1`)	69.1 (9.1)	50.3 (10.9)	51.4 (11.8)
**Age at diagnosis (years)**	45.3 (11.7)	36.7 (8.6)	58.33 (7.4)	35.2 (8.7)	32.8 (5.9)
**Duration of diabetes (years)**	9.9 (6.5)	14.1 (8.4)	10.7 (7.1)	15.0 (10.3)	18.6 (9.4)
**BMI (Kg/m** ^**2**^ **)**	33.9 (3.9)	32.1 (6.7)	28.3 (3.6)	38.7 (5.9)	26.8 (2.7)
**HOMA-IR**	16.3 (6.5)	8.1 (5.0)	3.0 (1.7)	3.6 (2.6)	2.3 (1.5)
**HOMA-B**	254 (267)	32 (19)	111 (157)	118 (111)	69 (43)
**FBG (mg/dL)**	151 (38)	238 (69)	114 (21)	115 (30)	113 (23)
**Fasting Insulin (mU/ml)**	43.1 (9.6)	13.5 (6.7)	10.3 (4.2)	12.1 (6.7)	8.3 (4.6)
**HbA1c (%)**	7.3 (0.9)	10.7 (2.3)	6.3 (0.7)	6.9 (0.8)	6.6 (0.9)

SIRD- severe insulin-resistant diabetes; SIDD- severe insulin deficient diabetes; MARD- mild age-related diabetes; MOD- mild obesity-related diabetes; MEOD- mild early-onset diabetes; BMI- body mass index; HOMA-IR- homeostatic model assessment for insulin resistance; HOMA-B- homeostatic model assessment of β-cell Function; FBG- fasting blood glucose; HbA1c- Haemoglobin A1c.

All values are shown as Mean (±Standard Deviation).

In Cluster 1 (SIRD), the primary dysfunction was a markedly increased insulin resistance associated with moderate obesity. The patients had a normal insulin secretory capacity and moderately abnormal glucose homeostasis. Most of the patients had peripheral neuropathy (Table 7).

**Table 7:** Diabetes comorbidities and complications in five T2D subtypes of 151 Emirati patients in the non-overlapping apices of distribution of clusters.

	SIRD (N = 25)	SIDD (N = 40)	MARD (N = 24)	MOD (N = 23)	MEOD (N = 39)	SIRD (N = 25)	P-value
**Retinopathy**	53 (35)	2 (8)	15 (37)	5 (20)	10 (43)	21 (53)	0.002
**Cataract**	12 (8)	1 (4)	3 (8)	2 (8)	0	6 (15)	0.241
**Glaucoma**	5 (3)	1 (4)	1 (3)	1 (4)	0	2 (5)	0.850
**CKD**	21 (13)	3 (12)	5 (12)	5 (20)	6 (26)	2 (5)	0.167
**CAD**	21 (14)	2 (8)	5 (12)	7 (29)	0	7 (18)	0.05
**History of Stroke**	9 (6)	1 (4)	3 (8)	3 (13)	0	2 (5)	0.451
**Hypertension**	86 (57)	18 (72)	25 (62)	16 (66)	14 (60)	13 (33)	0.013
**PAD**	10 (6)	1 (4)	2 (5)	1 (4)	3 (13)	3 (8)	0.663
**Peripheral Neuropathy**	88 (58)	12 (48)	23 (57)	13(54)	16 (69)	24 (61)	0.620

CKD- chronic kidney disease; CAD- coronary artery disease; PAD- peripheral artery disease

All values are shown as Number of patients (percentage)

In Cluster 2 (SIDD), the primary dysfunction was severe insulin secretory deficiency accompanied by high insulin resistance and the highest level of HbA1c. Patients were obese, with the most severe uncontrolled glucose homeostasis and a higher frequency of complications such as retinopathy, peripheral neuropathy, and ischemic heart disease (Table 7).

Cluster 3 (MARD) patients developed diabetes late in life and had the highest mean age at diagnosis. They were overweight and characterized by moderate insulin resistance, normal insulin secretory capacity, and mildly abnormal glucose homeostasis. However, these patients also had nephropathy, peripheral neuropathy, and ischemic heart disease (Table 7).

Patients in Cluster 4 (MOD) had the highest BMI and developed diabetes early in life. They were characterized by moderate insulin resistance but normal insulin secretory capacity, with mildly abnormal glucose homeostasis. However, these patients also had retinopathy and nephropathy (Table 7).

In Cluster 5 (MEOD), a novel T2D subtype, the patients were lean/overweight and developed the disease early in life. They had moderate insulin secretory dysfunction and mild insulin resistance with mildly abnormal glucose homeostasis (Table 5). These patients had retinopathy, peripheral neuropathy, and ischemic heart disease (Table 7).

## 4 Discussion

We attempted subtyping T2D in 348 Emirati patients using unsupervised soft cluster analysis by Auto Cluster IBM Modeler in the SPSS software, employing five etiological predictor variables: FBG, FSI, BMI, HbA1c and age at diagnosis. Multinomial logistic regression was used to validate the clustering process and to rank the importance of the predictor variables in each cluster. Five clusters were identified; the first four matched Ahlqvist et al [11] subgroups: SIRD, SIDD, MARD, and MOD. A fifth new subtype MEOD was identified in our dataset.

However, there was extensive overlap between clusters. Individuals in the non-overlapping apices of distribution of clusters were identified in only 151/348 patients (43%), with individuals appearing only once in a single cluster. The remaining 197/348 patients (57%) showed varying degrees of overlap, with individuals appearing in two or more clusters. As one lowers the silhouette coefficient values in a sliding scale below 1.0, the degree of overlap decreases and the number of individuals in the non-overlapping apices of clusters increases but their clinical homogeneity and discreetness drops. This extensive degree of overlap has been previously reported by different study groups: the Broad Institute of MIT [14, 17]; the Oxford Center of Diabetes, UK [15] and the Exeter Research Group, UK [16].

Ahlqvist et al. [11] employed a data-driven, unsupervised hard clustering method to identify mutually exclusive patient subgroups within large, newly diagnosed T2D cohorts. Their subtyping scheme, although replicated in multiple studies [9–13, 18–24]; including our current investigation, has been challenged by soft, unsupervised clustering techniques that revealed overlapping and alternative subgroupings [14–17]. Both approaches rely on continuous (non-discrete) clinical characteristics. Unlike discrete data with distinct categories, continuous data exists on a spectrum, hindering the definition of clear-cut cluster boundaries. Overlapping clusters inherently arise with such data, challenging the traditional concept of distinct, well-separated patient groups. Fuzzy boundaries further complicate cluster interpretation and labeling [14–17]. Hard unsupervised methods like Ahlqvist’s, rely on pre-defined subtypes, potentially overlooking unseen biological variations or dynamic processes. Conversely, soft unsupervised clustering avoids preconceived notions, potentially uncovering the true underlying data structure and heterogeneity. But, overlapping clusters, as observed in healthcare domains, can impede clinical decision-making due to ambiguous patient group assignment [31–33].

Previously, most T2D subtyping studies recruited newly diagnosed patients [9–17]. In contrast, in the present study, we tested the feasibility of clinical subtyping in T2D patients with long standing disease. The mean age of the patients was 56 years with mean diabetes duration exceeding 14 years. They were mostly obese, had various premorbid conditions, and had developed various complications of diabetes. In all five subtypes, the combination of the basic etiological dysfunction could still be identified despite co-morbidities and complications of the disease with advancing age. Our results agree with several studies where subtyping of T2D in long-standing disease have been successfully performed [3, 21–23, 26]. Despite temporal changes in lifestyle and environmental exposure causing decline in β-cell function and/or worsening of insulin resistance with increased frequency of complications, subtyping of long standing T2D is not obscured [21, 22, 24–26]. This is probably due to genetically determined factors that do not change over a lifetime.

To highlight the major etiological processes governing T2D subtypes, individuals in the non-overlapping apices of the cluster distribution (151/348) were selected to identify the major etiological determinants of a subtype. The four T2D subtypes, SIRD, SIDD, MARD, and MOD, which were identified in this cohort with long-standing disease, were mapped back to the four subtypes of newly diagnosed diabetes patients by Ahlqvist et al [11] in the Scania (ANDIS) study. Patients with SIRD and SIDD suffered severe abnormal glucose homeostasis, whereas patients with MARD and MOD had mild disease. The fifth type is a novel subtype of mild early onset T2D (MEOD) in mostly lean individuals.

In patients with SIRD (Cluster 1), the identified etiological dysfunction was severe peripheral insulin resistance. This is similar to the SIRD in the ANDIS study [11] and Group C in the IMI DIRECT study [15]. In patients with SIDD (Cluster 2), the identified etiological dysfunction was severe β-cell dysfunction, heightened by moderate/severe insulin resistance [29]. This subtype is similar to the SIDD described in the ANDIS Study (11) and the global archetype D, which had the worst glucose control, in the IMI-DIRECT study (15). In previous studies some patients with early-onset T2D had worse clinical outcomes and are at higher risk of stroke and myocardial infarction [34]. Patients with SIDD had early-onset and displayed characteristics of severe diabetes. Interestingly, the average age of onset for this subgroup (37.9 years) was comparable to the mild early-onset diabetes (MEOD) subgroup (36.5 years). However, the MEOD subgroup exhibited a distinctly milder clinical profile compared to the SIDD subgroup.

In patients with MARD (Cluster 3), the identified etiological dysfunction appeared to be mild peripheral resistance to insulin owing to advancing age. This subtype is similar to the MARD described in the ANDIS Study [11] and archetype A described in the IMI-DIRECT study [15]. In patients with MOD (Cluster 4), the identified etiological dysfunction was an obesity-driven peripheral resistance to insulin. This subtype is similar to the MOD described in the ANDIS Study [11] and archetype C described in the IMI-DIRECT study [15]. MEOD (Cluster 5) is a novel subtype that has not been previously reported in earlier studies. The patients were mostly lean, had early onset disease, mild/moderate β-cell dysfunction, and mild insulin resistance. This subtype of T2D has been previously identified in non-Caucasian ethnic groups in developing countries [35–37]. It is not surprising, therefore, that this cluster was identified among the Emiratis.

In summary, only the severe SIRD subtype appeared to be an independent disease entity. The statistical properties and clinical characteristics of the patients are distinct. Membership of most patients is restricted to this subtype and does not overlap with that of other subtypes. The next highest probability of being a distinct entity is the severe SIDD subtype. The other three mild subtypes, MARD, MORD, and MEOD did not qualify as independent disease entities. They exhibited an extensive overlap in subtype membership and high heterogeneity in their clinical characteristics.

The main aim of clustering is to identify patient subtypes with similar characteristics within a larger group of individuals with T2D, to enable clinicians to gain insights into the mechanisms of disease development and progression. This can potentially lead to personalized clinical management and improved patient outcomes. However, in our study, as in some other subtyping studies, it has been recognized that clustering based on continuous variables does not result in mutually exclusive subtypes [14–17, 32]. Therefore, integrating simplified, personalized metabolic profiles with clustering holds greater promise for guiding clinical decisions than subtyping alone [8, 32]. As per our results, the T2D specific phenotype profile: age at diagnosis, BMI, FBG, HbA1c, HOMA-B, and HOMA-IR could predict specific outcome for individual patients:

- Age at T2D diagnosis: Young age indicates a strong genetic predisposition. The younger the age at diagnosis, the more severe the disease and the higher the risk of complications. The older the age at diagnosis, the milder the disease [3].
- BMI: The higher the BMI, the higher the peripheral resistance to insulin, the more severe the disease, and the higher the risk of complications [38].
- FBG: The higher the Impaired Fasting Glucose level, the higher the hepatic insulin resistance and hepatic glucose production [1, 39–42].
- HbA1c: The higher the HbA1c, the greater the disease severity (2).
- HOMA-B: The lower the HOMA-B score, the more severe the β-cell dysfunction [30].
- HOMA-IR: The higher the HOMA-IR, the higher the whole-body peripheral resistance to insulin [30].

Our study has some limitations. We used FBG and FSI in the exploratory phase where cluster analysis was performed and HOMA indices in the explanatory phase where cluster outcomes and characteristics were identified. We acknowledge the presence of moderate collinearity between the HOMA indices used in the explanatory phase. This can lead to difficulty in separating the true effect of insulin resistance from the effect of insulin secretion. To mitigate these effects, we used principal component analysis introducing new uncorrelated variables from the original set. It is also important to note that the computer models generating these indices incorporate additional parameters such as glucagon secretion and liver glucose production. Furthermore, these indices continue to be used in diabetes research for the lack of perfect alternatives. We used them only for broader trends and cluster identification and not for precise rigorous measurements.

We also used FBG and FSI in the exploratory phase of analysis which is less concerned with individual variable effects. It focuses on pattern identification and data relationships without trying to isolate the impact of specific variables on an outcome. They are about finding the underlying structure, not establishing causal links. It does not suffer from collinearity of the variables used. Therefore, while the same parameters were used in both exploratory and explanatory techniques, collinearity is only a concern within the explanatory models and not between the two phases of analysis.

The strength of this study is in confirming that T2D subtyping can be performed at any stage of the disease. This provides insight into the stability and evolution of clusters. Although the number of patients is small, the study provided proof-of-principle that soft, unsupervised clustering techniques reveal overlapping subgroupings of T2D and uncover further aspects of heterogeneity of T2D [43]. Because this was a retrospective study, we relied on existing data collected from medical records and patient interviews, leading to a potential recall bias or missing information. The small number of participants, broad inclusion criteria, and potential bias in data selection may limit the generalizability of the findings. The long duration of illness and unmeasured or unknown confounders, such as diabetes complications and drug responses, make it difficult to establish a clear temporal relationship between exposure and outcome. Yet, despite all that noise, clusters of T2D could be identified.

## 5 Conclusions

Despite the complex picture of long-standing T2D with comorbidities, complications and varied therapy, our study demonstrates the feasibility of identifying subtypes and their underlying causes. Five clusters were identified: the first four matched Ahlqvist et al [11] subgroups. Two subtypes were characterized by severe disease and two by mild disease. A fifth novel subtype, identified among mostly lean individuals is usually seen in non-white populations. While clustering provides valuable insights into the architecture of T2D subtypes, its application to individual patient management would remain limited due to overlapping characteristics. Therefore, integrating simplified, personalized metabolic profiles with clustering holds greater promise for guiding clinical decisions than subtyping alone. Future studies on the pathogenesis of subtypes and the prognosis of drug therapy are needed. Further longitudinal investigations are also required to clarify subtype stability over time, elucidate the factors influencing transitions between subtypes, and translate these findings into concrete clinical applications.

## Supporting information

S1 Checklist Human participants research checklist. (PDF)

S1 Table Heat map of probabilities of cluster membership showing overlap between clusters. A Silhouette Index of 1.0 indicates no overlap and <1.0 indicates overlap between clusters.
(DOCX)

S2 Table The contribution of the five primary independent variables (Age at diagnosis, BMI, fasting insulin, Fasting Blood Glucose (FBG) and HbA1c) as predictors for T2D clusters using multinomial logistic regression. (DOCX)

S1 File SPSS_T2D_Database-Bayoumi. (SAV)
