# Supplementary material for: Etiologies underlying subtypes of long-standing type 2 diabetes
Source: PLoS One. 2024 May 28;19(5):e0304036. doi: 10.1371/journal.pone.0304036 (PMC11132508; doi:10.1371/journal.pone.0304036)
Supplement: S1 Checklist — (PDF) [file pone.0304036.s001.pdf]

Human Participants Research Checklist

Complete the following if your study involved human participants or human participants' data. These questions should be addressed for prospective and retrospective studies.

1. Did you obtain ethics approval for this study? YES

   - If yes, please upload (file type "Other") the original approval document you received from your ethics committee. If the original document is in another language, please also provide an English translation.

   Document Uploaded:

   1. Submitted IRB application [Attached].
   2. IRB approval from Mohammed Bin Rashid University, Dubai, UAE, dated 14th January 2020 [Attached].
   3. IRB approval from Dubai Health Authority, Dubai, UAE, dated 23rd January 2020 [Attached].

   If you did not obtain ethical approval, please explain why this was not required below.

2. If you prospectively recruited human participants for the study – for example, you conducted a clinical trial, distributed questionnaires, or obtained tissues, data or samples for the purposes of this study, please report in the Methods:

   i. The recruitment period for this study started on 24th January 2020 and ended 31st December 2022.[Included in Methods Section].

   ii. whether participants provided informed consent, and if so, what type was obtained (for instance, written or verbal, and if verbal, how it was documented and witnessed). If your study included minors, state whether you obtained consent from parents or guardians. If the need for consent was waived by the ethics committee, please include this information.

   Written informed Consent was obtained from every patient [Document attached]. No minors were included.

3. If you are reporting a retrospective study of medical records or archived samples, please report in the Methods section:

   i. The day, month and year when the data were accessed for research purposes. From 24th January 2020 up to 31st December 2022 data from Hospital Information System [SALAMA] was obtained from Dubai Health Authority. [Included in Methods Section].

   ii. Whether authors had access to information that could identify individual participants during or after data collection

4. Only the PI had the KEY linking data to patients. The KEY is kept secure under lock. None of the authors had access to information that could identify individual participants during or after data collection.
