# Supplementary material for: Etiologies underlying subtypes of long-standing type 2 diabetes
Source: PLoS One. 2024 May 28;19(5):e0304036. doi: 10.1371/journal.pone.0304036 (PMC11132508; doi:10.1371/journal.pone.0304036)
Supplement: S1 Table — A Silhouette Index of 1.0 indicates no overlap and <1.0 indicates overlap between clusters. (DOCX) [file pone.0304036.s002.docx]

**S1 Table: Heat map of probabilities of cluster membership showing overlap between clusters.** A Silhouette Index of 1.0 indicates no overlap and <1.0 indicates overlap between clusters.

| **Member­­_ID** | **Cluster 1** | **Cluster 2** | **Cluster 3** | **Cluster 4** | **Cluster 5** |
| --- | --- | --- | --- | --- | --- |
| **Cluster 1** |  |  |  |  |  |
| DDC50044 | 0.81 | 0.01 | 0.00 | 0.19 | 0 |
| DDC50130 | 0.89 | 0.11 | 0 | 0 | 0 |
| **Cluster 2** |  |  |  |  |  |
| DH00004 | .01 | .99 | .00 | .00 | .00 |
| DDC50276 | .00 | 1.00 | .00 | .00 | .00 |
| DDC50202 | .00 | 1.00 | .00 | .00 | .00 |
| DDC50084 | .90 | .00 | .90 | .00 | .00 |
| DDC50029 | .00 | .75 | .00 | .25 | .00 |
| DH00159 | .00 | .92 | .00 | .00 | .08 |
| DDC50274 | .00 | .66 | .00 | .00 | .34 |
| DDC50331 | .00 | .66 | .00 | .00 | .34 |
| DH00186 | .00 | .66 | .00 | .00 | .34 |
| DDC50183 | .00 | .75 | .00 | .25 | .00 |
| DH00162 | .00 | .92 | .00 | .00 | .08 |
| DDC50192 | .00 | .66 | .00 | .00 | .34 |
| DDC50138 | .00 | .83 | .00 | .17 | .00 |
| DH00192 | .00 | .64 | .00 | .00 | .36 |
| DDC50127 | .00 | .92 | .00 | .00 | .08 |
| DDC50133 | .00 | .92 | .00 | .00 | .08 |
| DH00186 | .00 | .66 | .00 | .00 | .34 |
| **Cluster 3** |  |  |  |  |  |
| DDC50179 | 0.12 | 0 | 0.79 | 0.09 | 0 |
| DDC50124 | 0.12 | 0 | 0.79 | 0.09 | 0 |
| DH00102 | 0.04 | 0 | 0.96 | 0 | 0 |
| DDC50078 | 0 | 0 | 0.92 | 0.08 | 0 |
| DDC50093 | 0 | 0 | 0.9 | 0.1 | 0 |
| DDC50270 | 0 | 0 | 0.93 | 0.08 | 0 |
| DDC50307 | 0 | 0 | 0.81 | 0.19 | 0 |
| DDC50278 | 0 | 0 | 0.76 | 0.24 | 0 |
| DDC50119 | 0 | 0 | 0.97 | 0.03 | 0 |
| DDC50170 | 0 | 0.02 | 0.82 | 0.16 | 0 |
| DH00185 | 0 | 0 | 0.97 | 0.03 | 0 |
| DDC50107 | 0 | 0 | 0.91 | 0.09 | 0 |
| DDC50262 | 0 | 0.26 | 0.74 | 0 | 0 |
| DDC50034 | 0 | 0 | 0.99 | 0.01 | 0 |
| DDC50237 | 0 | 0 | 0.97 | 0.03 | 0 |
| DDC50182 | 0 | 0.09 | 0.58 | 0.33 | 0 |
| DDC50210 | 0 | 0 | 0.81 | 0.19 | 0 |
| DDC50164 | 0 | 0 | 1 | 0 | 0 |
| DDC50351 | 0 | 0 | 0.97 | 0.03 | 0 |
| DDC50223 | 0 | 0 | 0.9 | 0.1 | 0 |
| DDC50144 | 0 | 0 | 0.91 | 0.09 | 0 |
| DDC50150 | 0 | 0 | 1 | 0 | 0 |
| DDC50295 | 0 | 0 | 0.97 | 0.03 | 0 |
| DH00181 | 0 | 0 | 0.81 | 0.19 | 0 |
| DDC50340 | 0 | 0 | 0.94 | 0.07 | 0 |
| DDC50005 | 0 | 0 | 1 | 0.01 | 0 |
| DDC50108 | 0 | 0 | 1 | 0 | 0 |
| DDC50074 | 0 | 0 | 0.98 | 0.02 | 0 |
| DDC50248 | 0 | 0 | 0.74 | 0.26 | 0 |
| DH00158 | 0 | 0 | 1 | 0 | 0 |
| DH00178 | 0 | 0 | 0.9 | 0.1 | 0 |
| DDC50293 | 0 | 0 | 0.97 | 0.03 | 0 |
| DDC50246 | 0 | 0.02 | 0.98 | 0 | 0 |
| DDC50032 | 0 | 0 | 0.98 | 0.02 | 0 |
| DDC50205 | 0 | 0 | 0.97 | 0.03 | 0 |
| DH00240 | 0 | 0 | 1 | 0 | 0 |
| DDC50134 | 0 | 0.01 | 0.62 | 0.38 | 0 |
| DDC50312 | 0 | 0 | 0.97 | 0.03 | 0 |
| DH00128 | 0 | 0 | 0.96 | 0.04 | 0 |
| DDC50117 | 0 | 0.01 | 0.62 | 0.38 | 0 |
| DDC50287 | 0 | 0 | 0.96 | 0.04 | 0 |
| DH00091 | 0 | 0 | 0.99 | 0.01 | 0 |
| DDC50116 | 0 | 0 | 0.53 | 0 | 0.47 |
| DDC50280 | 0 | 0.01 | 0.99 | 0 | 0 |
| DDC50244 | 0 | 0 | 0.53 | 0 | 0.47 |
| DDC50137 | 0 | 0 | 0.53 | 0 | 0.47 |
| DDC50013 | 0 | 0.03 | 0.97 | 0 | 0 |
| DDC50219 | 0 | 0 | 0.53 | 0 | 0.47 |
| DDC50216 | 0 | 0.01 | 0.99 | 0 | 0 |
| DDC50260 | 0 | 0.01 | 0.99 | 0 | 0 |
| DDC50229 | 0 | 0.01 | 0.99 | 0 | 0 |
| DDC50308 | 0 | 0 | 0.53 | 0 | 0.47 |
| DDC50159 | 0 | 0 | 0.53 | 0 | 0.47 |
| DH00220 | 0 | 0 | 1 | 0 | 0 |
| DDC50199 | 0 | 0 | 0.53 | 0 | 0.47 |
| DH00179 | 0 | 0 | 0.53 | 0 | 0.47 |
| DDC50188 | 0 | 0.02 | 0.98 | 0 | 0 |
| DDC50302 | 0 | 0 | 0.53 | 0 | 0.47 |
| DDC50072 | 0 | 0 | 0.53 | 0 | 0.47 |
| DDC50021 | 0 | 0.01 | 0.99 | 0 | 0 |
| DDC50177 | 0 | 0 | 1 | 0 | 0 |
| DDC50061 | 0 | 0.1 | 0.9 | 0 | 0 |
| **Cluster 4** |  |  |  |  |  |
| DDC50241 | 0.04 | 0 | 0 | 0.96 | 0 |
| DDC50277 | 0.1 | 0 | 0 | 0.9 | 0 |
| DDC50319 | 0.02 | 0 | 0 | 0.98 | 0 |
| DDC50142 | 0.02 | 0 | 0 | 0.98 | 0 |
| DDC50339 | 0 | 0.13 | 0 | 0.6 | 0.28 |
| DDC50085 | 0 | 0.03 | 0 | 0.97 | 0 |
| DH00161 | 0 | 0.07 | 0 | 0.93 | 0 |
| DH00167 | 0 | 0.42 | 0 | 0.58 | 0 |
| DDC50322 | 0 | 0 | 0.32 | 0.68 | 0 |
| DH00184 | 0 | 0.25 | 0 | 0.66 | 0.09 |
| DDC50031 | 0 | 0.01 | 0 | 0.58 | 0.42 |
| DH00202 | 0 | 0.01 | 0 | 0.99 | 0 |
| DDC50343 | 0 | 0 | 0 | 1 | 0 |
| DDC50334 | 0 | 0 | 0.32 | 0.68 | 0 |
| DDC50155 | 0 | 0 | 0 | 1 | 0 |
| DH00005 | 0 | 0 | 0 | 1 | 0 |
| DH00189 | 0 | 0 | 0.32 | 0.68 | 0 |
| DDC50181 | 0 | 0.02 | 0 | 0.82 | 0.17 |
| DDC50303 | 0 | 0.08 | 0.37 | 0.55 | 0 |
| DDC50158 | 0 | 0.01 | 0 | 0.99 | 0 |
| DDC50135 | 0 | 0.01 | 0.01 | 0.99 | 0 |
| DDC50207 | 0 | 0 | 0.06 | 0.94 | 0 |
| DH00166 | 0 | 0 | 0.02 | 0.98 | 0 |
| DDC50132 | 0 | 0.05 | 0 | 0.95 | 0 |
| DDC50245 | 0 | 0 | 0.02 | 0.98 | 0 |
| DDC50325 | 0 | 0.01 | 0.01 | 0.99 | 0 |
| DDC50113 | 0 | 0.02 | 0.04 | 0.94 | 0 |
| DDC50327 | 0 | 0 | 0 | 1 | 0 |
| DDC50096 | 0 | 0.24 | 0 | 0.76 | 0 |
| DDC50265 | 0 | 0.02 | 0.04 | 0.94 | 0 |
| DDC50306 | 0 | 0 | 0.02 | 0.99 | 0 |
| DDC50313 | 0 | 0.07 | 0 | 0.93 | 0 |
| DH00040 | 0 | 0 | 0 | 1 | 0 |
| DDC50129 | 0 | 0.01 | 0.01 | 0.99 | 0 |
| DDC50168 | 0 | 0 | 0.02 | 0.98 | 0 |
| DDC50045 | 0 | 0 | 0 | 1 | 0 |
| DDC50103 | 0 | 0.06 | 0.01 | 0.93 | 0 |
| DH00183 | 0 | 0 | 0.36 | 0.64 | 0 |
| DDC50284 | 0 | 0.05 | 0 | 0.95 | 0 |
| DDC50329 | 0 | 0 | 0 | 1 | 0 |
| DDC50047 | 0 | 0.01 | 0.01 | 0.99 | 0 |
| DDC50086 | 0 | 0.32 | 0 | 0.69 | 0 |
| DDC50255 | 0 | 0.24 | 0 | 0.76 | 0 |
| DDC50090 | 0 | 0.02 | 0 | 0.98 | 0 |
| DDC50304 | 0 | 0.06 | 0.01 | 0.93 | 0 |
| DDC50231 | 0 | 0 | 0 | 1 | 0 |
| DDC50190 | 0 | 0.01 | 0.01 | 0.99 | 0 |
| DDC50311 | 0 | 0 | 0 | 1 | 0 |
| DDC50203 | 0 | 0 | 0.02 | 0.98 | 0 |
| DDC50335 | 0 | 0 | 0.15 | 0.85 | 0 |
| **Cluster 5** |  |  |  |  |  |
| DDC50309 | 0 | 0 | 0 | 0.42 | 0.58 |
| DDC50172 | 0 | 0 | 0 | 0.12 | 0.88 |
| DDC50148 | 0 | 0 | 0 | 0.01 | 0.99 |
| DDC50208 | 0 | 0.13 | 0 | 0.12 | 0.76 |
| DDC50344 | 0 | 0 | 0 | 0.12 | 0.88 |
| DDC50256 | 0 | 0 | 0 | 0.32 | 0.68 |
| DDC50209 | 0 | 0.03 | 0 | 0.25 | 0.72 |
| DDC50128 | 0 | 0 | 0 | 0.01 | 0.99 |
| DDC50154 | 0 | 0.01 | 0 | 0 | 0.99 |
| DDC50200 | 0 | 0.24 | 0 | 0 | 0.76 |
| DDC50261 | 0 | 0.02 | 0.12 | 0 | 0.86 |
| DDC50173 | 0 | 0.25 | 0 | 0 | 0.75 |
| DDC50206 | 0 | 0 | 0.09 | 0 | 0.91 |
| DDC50204 | 0 | 0.01 | 0 | 0 | 0.99 |
| DDC50097 | 0 | 0 | 0 | 0 | 1 |
| DDC50011 | 0 | 0 | 0.09 | 0 | 0.91 |
| DDC50165 | 0 | 0 | 0.3 | 0 | 0.7 |
| DDC50174 | 0 | 0.04 | 0 | 0 | 0.96 |
| DDC50092 | 0 | 0 | 0.09 | 0 | 0.91 |
| DDC50075 | 0 | 0.42 | 0 | 0 | 0.58 |
| DDC50299 | 0 | 0.42 | 0 | 0 | 0.58 |
| DDC50121 | 0 | 0.02 | 0.12 | 0 | 0.86 |
| DH00039 | 0 | 0 | 0.26 | 0 | 0.74 |
| DDC50050 | 0 | 0 | 0.24 | 0 | 0.76 |
| DDC50283 | 0 | 0 | 0 | 0 | 1 |
| DDC50253 | 0 | 0.02 | 0.12 | 0 | 0.86 |
| DDC50281 | 0 | 0.02 | 0.12 | 0 | 0.86 |
| DDC50042 | 0 | 0 | 0 | 0 | 1 |
| DDC50224 | 0 | 0 | 0 | 0 | 1 |
| DH00151 | 0 | 0.01 | 0 | 0 | 0.99 |
| DDC50315 | 0 | 0.04 | 0.06 | 0 | 0.9 |
| DDC50250 | 0 | 0 | 0 | 0 | 1 |
| DDC50083 | 0 | 0.02 | 0.12 | 0 | 0.86 |
| DDC50222 | 0 | 0 | 0.26 | 0 | 0.74 |
| DDC50289 | 0 | 0.02 | 0 | 0 | 0.98 |
| DDC50166 | 0 | 0.01 | 0 | 0 | 0.99 |
| DDC50290 | 0 | 0.02 | 0.12 | 0 | 0.86 |
| DDC50211 | 0 | 0.04 | 0 | 0 | 0.96 |
| DDC50169 | 0 | 0 | 0.26 | 0 | 0.74 |
| DDC50235 | 0 | 0.01 | 0.04 | 0 | 0.95 |
| DDC50115 | 0 | 0.02 | 0.12 | 0 | 0.86 |
| DDC50341 | 0 | 0 | 0.09 | 0 | 0.91 |
| DDC50234 | 0 | 0.02 | 0 | 0 | 0.98 |
| DDC50089 | 0 | 0.01 | 0 | 0 | 0.99 |
| DDC50267 | 0 | 0 | 0.09 | 0 | 0.91 |
| DDC50226 | 0 | 0.04 | 0 | 0 | 0.96 |
| DDC50279 | 0 | 0 | 0.09 | 0 | 0.91 |
| DDC50149 | 0 | 0 | 0 | 0 | 1 |
| DDC50197 | 0 | 0 | 0 | 0 | 1 |
| DDC50275 | 0 | 0.02 | 0.12 | 0 | 0.86 |
| DDC50120 | 0 | 0 | 0.11 | 0 | 0.89 |
| DDC50217 | 0 | 0 | 0.24 | 0 | 0.76 |
| DDC50242 | 0 | 0.04 | 0 | 0 | 0.96 |
| DH00229 | 0 | 0.04 | 0 | 0 | 0.96 |
| DDC50171 | 0 | 0 | 0.09 | 0 | 0.91 |
| DDC50099 | 0 | 0 | 0.26 | 0 | 0.74 |
| DDC50076 | 0 | 0 | 0.26 | 0 | 0.74 |
| DDC50114 | 0 | 0 | 0.26 | 0 | 0.74 |
| DDC50162 | 0 | 0 | 0 | 0 | 1 |
| DDC50298 | 0 | 0.02 | 0.12 | 0 | 0.86 |
| DDC50323 | 0 | 0 | 0.24 | 0 | 0.76 |
| DDC50228 | 0 | 0 | 0.09 | 0 | 0.91 |
| DDC50151 | 0 | 0 | 0.26 | 0 | 0.74 |
| DDC50185 | 0 | 0 | 0.24 | 0 | 0.76 |
| DDC50239 | 0 | 0 | 0.3 | 0 | 0.7 |
| DDC50160 | 0 | 0.02 | 0.12 | 0 | 0.86 |
