# Supplementary material for: Etiologies underlying subtypes of long-standing type 2 diabetes
Source: PLoS One. 2024 May 28;19(5):e0304036. doi: 10.1371/journal.pone.0304036 (PMC11132508; doi:10.1371/journal.pone.0304036)
Supplement: S2 Table — (DOCX) [file pone.0304036.s003.docx]

| **S2 Table: The contribution of the five primary independent variables (Age at diagnosis, BMI, Fasting Insulin, Fasting Blood Glucose (FBG) and HbA1c) as predictors for T2D clusters using multinomial logistic regression.** | | | | | | | | | | | | | |  |  |  |  |
| --- | --- | --- | --- | --- | --- | --- | --- | --- | --- | --- | --- | --- | --- | --- | --- | --- | --- |
| There are 1392 (80.0%) cells (i.e., dependent variable levels by subpopulations) with zero frequencies. | | | | | | | | | | | | | |  |  |  |  |
| Measures of Monotone Association table is not generated because the dependent variable does not have exactly two levels. | | | | | | | | | | | | | |  |  |  |  |
| 1. **Case Processing Summary** | | | | | | | | | | | | |  |  |  |  |  |
|  | | | | | | N | | | Marginal Percentage | | | |  |  |  |  |  |
| Cluster5 | | cluster-1 | | | | 27 | | | 7.8% | | | |  |  |  |  |  |
|  |  | cluster-2 | | | | 57 | | | 16.4% | | | |  |  |  |  |  |
|  |  | cluster-3 | | | | 86 | | | 24.7% | | | |  |  |  |  |  |
|  |  | cluster-4 | | | | 73 | | | 21.0% | | | |  |  |  |  |  |
|  |  | cluster-5 | | | | 105 | | | 30.2% | | | |  |  |  |  |  |
| Valid | | | | | | 348 | | | 100.0% | | | |  |  |  |  |  |
| Missing | | | | | | 0 | | |  | | | |  |  |  |  |  |
| Total | | | | | | 348 | | |  | | | |  |  |  |  |  |
| Subpopulation | | | | | | 348^a^ | | |  | | | |  |  |  |  |  |
| a. The dependent variable has only one value observed in 348 (100.0%) subpopulations. | | | | | | | | | | | | |  |  |  |  |  |
| 1. **Model Fitting Information** | | | | | | | | | | | | | | | | | |
| Model | | | | Model Fitting Criteria | | | | | | | | | | Likelihood Ratio Tests | | | |
|  |  |  |  | AIC | | | | BIC | | | -2 Log Likelihood | | | Chi-Square | | df | Sig. |
| Intercept Only | | | | 1072.363 | | | | 1087.772 | | | 1064.363 | | |  | |  |  |
| Final | | | | 283.849 | | | | 376.302 | | | 235.849 | | | 828.514 | | 20 | <.001 |
| 1. **Goodness-of-Fit** | | | | | | | | | | | |  |  |  |  |  |  |
|  | | | Chi-Square | | | | df | | | Sig. | |  |  |  |  |  |  |
| **Pearson** | | | **48812.282** | | | | **1368** | | | **.000** | |  |  |  |  |  |  |
| Deviance | | | 235.849 | | | | 1368 | | | 1.000 | |  |  |  |  |  |  |
| 1. **Pseudo R-Square** | | | | | | | |  |  |  |  |  |  |  |  |  |  |
| Cox and Snell | | | | | .908 | | |  |  |  |  |  |  |  |  |  |  |
| Nagelkerke | | | | | .952 | | |  |  |  |  |  |  |  |  |  |  |
| McFadden | | | | | .778 | | |  |  |  |  |  |  |  |  |  |  |

1. **Likelihood Ratio Tests**
2. **Parameter Estimates**

| Parameter Estimates | | | | | | | | | |
| --- | --- | --- | --- | --- | --- | --- | --- | --- | --- |
| Cluster5^a^ | | B | Std. Error | Wald | df | Sig. | Exp(B) | 95% Confidence Interval for Exp(B) | |
|  |  |  |  |  |  |  |  | Lower Bound | Upper Bound |
| cluster-1 | Intercept | -62.413 | 11.189 | 31.115 | 1 | <.001 |  |  |  |
|  | BMI | .741 | .158 | 22.031 | 1 | <.001 | 2.099 | 1.540 | 2.860 |
|  | FBG | .012 | .019 | .387 | 1 | .534 | 1.012 | .975 | 1.050 |
|  | HbA1C | -.201 | .720 | .078 | 1 | .781 | .818 | .199 | 3.356 |
|  | AgeAtDiagnosis | .355 | .095 | 13.902 | 1 | <.001 | 1.426 | 1.183 | 1.719 |
|  | Insulin_DH | .904 | .201 | 20.134 | 1 | <.001 | 2.469 | 1.664 | 3.663 |
| cluster-2 | Intercept | -54.489 | 8.221 | 43.936 | 1 | <.001 |  |  |  |
|  | BMI | .323 | .105 | 9.462 | 1 | .002 | 1.381 | 1.124 | 1.697 |
|  | FBG | .092 | .019 | 23.147 | 1 | <.001 | 1.096 | 1.056 | 1.138 |
|  | HbA1C | 2.823 | .516 | 29.941 | 1 | <.001 | 16.828 | 6.122 | 46.260 |
|  | AgeAtDiagnosis | .056 | .052 | 1.168 | 1 | .280 | 1.057 | .956 | 1.170 |
|  | Insulin_DH | .222 | .067 | 10.869 | 1 | <.001 | 1.249 | 1.094 | 1.425 |
| cluster-3 | Intercept | -19.345 | 4.254 | 20.677 | 1 | <.001 |  |  |  |
|  | BMI | .246 | .087 | 8.050 | 1 | .005 | 1.279 | 1.079 | 1.515 |
|  | FBG | -.014 | .010 | 2.065 | 1 | .151 | .986 | .967 | 1.005 |
|  | HbA1C | -.809 | .412 | 3.857 | 1 | .050 | .446 | .199 | .998 |
|  | AgeAtDiagnosis | .385 | .052 | 54.652 | 1 | <.001 | 1.470 | 1.327 | 1.628 |
|  | Insulin_DH | .206 | .046 | 20.368 | 1 | <.001 | 1.228 | 1.123 | 1.343 |
| cluster-4 | Intercept | -31.145 | 4.547 | 46.917 | 1 | <.001 |  |  |  |
|  | BMI | .834 | .109 | 58.239 | 1 | <.001 | 2.302 | 1.858 | 2.851 |
|  | FBG | .008 | .009 | .816 | 1 | .366 | 1.008 | .991 | 1.026 |
|  | HbA1C | .183 | .296 | .381 | 1 | .537 | 1.201 | .672 | 2.146 |
|  | AgeAtDiagnosis | .022 | .038 | .331 | 1 | .565 | 1.022 | .949 | 1.101 |
|  | Insulin_DH | .113 | .044 | 6.682 | 1 | .010 | 1.120 | 1.028 | 1.220 |
| a. The reference category is: cluster-5. | | | | | | | | | |

1. **Classification**

| Classification | | | | | | |
| --- | --- | --- | --- | --- | --- | --- |
| Observed | Predicted | | | | | |
|  | cluster-1 | cluster-2 | cluster-3 | cluster-4 | cluster-5 | Percent Correct |
| cluster-1 | 25 | 1 | 1 | 0 | 0 | 92.6% |
| cluster-2 | 0 | 51 | 1 | 2 | 3 | 89.5% |
| cluster-3 | 1 | 0 | 75 | 3 | 7 | 87.2% |
| cluster-4 | 1 | 1 | 3 | 64 | 4 | 87.7% |
| cluster-5 | 0 | 4 | 6 | 7 | 88 | 83.8% |
| Overall Percentage | 7.8% | 16.4% | 24.7% | 21.8% | 29.3% | 87.1% |
